# Viral hepatitis and hepatocellular carcinoma

**DOI:** 10.1186/1477-7819-3-27

**Published:** 2005-05-20

**Authors:** Peter P Michielsen, Sven M Francque, Jurgen L van Dongen

**Affiliations:** 1 Division of Gastroenterology and Hepatology University Hospital Antwerp, Belgium

## Abstract

**Background:**

Hepatocellular carcinoma (HCC) is one of the most common malignant tumors in the world. The incidence of HCC varies considerably with the geographic area because of differences in the major causative factors. Chronic hepatitis B and C, mostly in the cirrhotic stage, are responsible for the great majority of cases of HCC worldwide. The geographic areas at the highest risk are South-East Asia and sub-Saharan Africa, here hepatitis B is highly endemic and is the main cause of HCC. In areas with an intermediate rate of HCC such as Southern Europe and Japan, hepatitis C is the predominant cause, whereas in low rate areas such as Northern Europe and the USA, HCC is often related to other factors as alcoholic liver disease. There is a rising incidence in HCC in developed countries during the last two decades, due to the increasing rate of hepatitis C infection and improvement of the clinical management of cirrhosis.

**Methods:**

This article reviews the literature on hepatitis and hepatocellular carcinoma. The Medline search was carried out using these key words and articles were selected on epidemiology, risk factors, screening, and prevention of hepatocellular carcinoma.

**Results:**

Screening of patients with advanced chronic hepatitis B and C with hepatic ultrasound and determination of serum alfa-fetoprotein may improve the detection of HCC, but further studies are needed whether screening improves clinical outcome.

Hepatitis B and C viruses (HBV/HCV) can be implicated in the development of HCC in an indirect way, through induction of chronic inflammation, or directly by means of viral proteins or, in the case of HBV, by creation of mutations by integration into the genome of the hepatocyte.

**Conclusion:**

The most effective tool to prevent HCC is avoidance of the risk factors such as viral infection. For HBV, a very effective vaccine is available. Preliminary data from Taiwan indicate a protective effect of universal vaccination on the development of HCC. Vaccination against HBV should therefore be a health priority. In patients with chronic hepatitis B or C, interferon-alfa treatment in a noncirrhotic stage is protective for HCC development in responders, probably by prevention of cirrhosis development. When cirrhosis is already present, the protective effect is less clear. For cirrhosis due to hepatitis B, a protective effect was demonstrated in Oriental, but not in European patients. For cirrhosis due to hepatitis C, interferon-alfa treatment showed to be protective in some studies, especially in Japan with a high incidence of HCC in untreated patients. Virological, but also merely biochemical response, seems to be associated with a lower risk of development of HCC. As most studies are not randomized controlled trials, no definitive conclusions on the long-term effects of interferon-alfa in HBV or HCV cirrhosis can be established. Especially in hepatitis C, prospective studies should be performed using the more potent reference treatments for cirrhotics, namely the combination of peginterferon and ribavirin.

## Epidemiology of hepatocellular carcinoma

### Background

Hepatocellular carcinoma (HCC) is one of the most common malignant tumors, representing more than 5% of all cancers. The estimated annual number of cases exceeds 500,000 [1], with a mean annual incidence of around 3–4% [2]. In terms of relative frequencies, HCC ranks as the fifth most common cancer in the world, it is also the fifth among men and eighth among women; it is the second among cancers of the digestive tract after stomach cancer [3].

The incidence of HCC varies considerably with the geographic area because of differences in the major causative factors. The geographic areas at the highest risk are located in Eastern Asia, with age-adjusted incidence rates (AAIR) ranging from 27.6 to 36.6 per 100,000 in men; Middle Africa (AAIR 20.8–31.1/100,000) and some Western African countries (30–48/100,000). The geographic areas at lowest risk are Northern Europe, Australia, New Zealand and the Caucasian populations of North and Latin America (AAIR 1.5–3.0). In Southern Europe, AAIR is around 10 per 100,000 in men [3].

The most powerful risk factor for development of HCC is the existence of liver cirrhosis, regardless of its etiology [4]. Among cirrhotics, viral infection and high alcohol intake are associated with the highest risk [5-8].

Of the primary hepatitis viruses, only hepatitis B and C viruses cause HCC [9]. Hepatitis A and E viruses do not produce long-term pathological sequelae. Although hepatitis D virus (HDV) always occurs as co-infection with hepatitis B virus and leads to severe acute or chronic hepatic disease, there is controversy whether it increases the carcinogenic potential [10,11].

### Risk factors for development of HCC

#### Hepatitis B

Hepatitis B virus (HBV) infection is a major public health problem. It is estimated that two billion people have been infected worldwide and 360 million suffer from chronic HBV infection [12]. Over 520,000 die each year, 50,000 from acute hepatitis B, 470,000 from cirrhosis and liver cancer. In South-East Asia hepatitis B is mostly acquired perinatally from an infected mother. In sub-Saharan Africa, it is mostly acquired in early childhood by horizontal infection, whereas in Northwestern Europe, North America and Australia infection is mainly through sexual contact or needle sharing among injecting drug users, with a peak incidence in the 15–25 age group [12]. Infection acquired perinatally and in early childhood is usually asymptomatic, becoming chronic in 90 and 30% of cases, respectively. In adults, infection resolves in >95% with loss of serum HBsAg and the appearance of anti-HBs. Chronic infection is characterized by the persistence of HBsAg for more than 6 months. Acute hepatitis B usually results in complete recovery with little if any risk of HCC. In cases with persistent HBV infection, HBV is one of the most important risk factors for HCC.

Chronic HBV infection presents as one of three potentially successive phases: *immune tolerant*, *immune active *and *low- or non-replicative*. In the *immune tolerant phase*, serum HBsAg and HBeAg are detectable, serum HBV DNA levels are high, serum aminotransferases are normal or minimally elevated. In the *immune active phase*, serum HBV DNA levels decrease and serum aminotransferase levels increase. Flares of aminotransferases may be observed, in some patients these flares are followed by HBeAg-anti-HBe seroconversion. Following this conversion, in the *low*- *or non-replicative phase *the HBV replication persists but at a very low level suppressed by the host immune response. HBV DNA in serum is undetectable by conventional, non-PCR based techniques. This phase is also called the '*inactive carrier state*'. It may lead to resolution of HBV infection where HBsAg becomes undetectable and anti-HBs is detected, anti-HBc staying positive as sign of contact with the virus. Recently it has been reported that HBV DNA can persist in the serum and liver tissue even after negativation of HBsAg [13]. Recent advances in molecular technology have allowed the isolation of HBV variants that either cannot produce HBeAg or produce it less efficiently, based on precore stop codon mutation and mutations in the core promoter region respectively. In patients with HBV variants, progressive liver damage occurs in parallel with relatively high levels of viremia. In perinatally infected people, the immunotolerant phase lasts till the age of 15–35 years, after which hepatitis flares may occur, leading eventually to viral remission. In patients infected during later childhood or adulthood, there is no immunotolerant phase.

Most studies on the risk of developing HCC in chronic HBV infection have been performed in the Far East. Here, most patients acquired the HBV infection as newborn infants [14]. It has been noted that the probability of acquiring HCC increases with severity of liver disease. The annual risk of HCC is 0.5% for asymptomatic HBsAg carriers and 0.8% for patients with chronic hepatitis B [15]. Patients with HBV-cirrhosis have a 1000 times higher risk of developing HCC compared to a HBsAg negative control group [16]. The incidence of HCC in compensated cirrhosis due to HBV from Asia was 2.7%. In Japan, the mean interval between the time of initial infection with HBV and the occurrence of HCC is 50 years. As most people here are infected at birth, HBV related liver cirrhosis usually develops in patients in their 40's and HCC in their 50's [17].

Few adequate studies have been performed in the West to address the issue of the incidence of HCC in persons who are positive for HBsAg. Most of the studies in Western countries have included small numbers of HBsAg positive patients and/or have not specifically analyzed the group of HBsAg carriers. There is also lack of uniformity in the timing of initiation of follow-up monitoring. In a cohort of 350 Western European patients with compensated cirrhosis followed for a mean period of 6 years, the 5-year cumulative incidence of HCC was 6% [18,19]. The incidence was 2.2% in a series of 179 untreated Caucasian patients [19,20]. In a retrospective analysis of cirrhotic European patients with HBV infection, the 5-year incidence of HCC was 9% irrespective of HBeAg or HBV DNA status at the time of diagnosis of cirrhosis [21].

The hepatitis B replication status seems to play an important role in determining the risk of development of HCC [22-24]. A recent study found that whereas the relative risk of HCC among men with HBsAg alone was 9.6 compared to those without HBsAg, the risk increased to 60.2 when they were positive for both HBsAg and HBeAg [23]. Another analysis showed that the level of HBV DNA is a prognostic marker for HBV-related HCC and that HCC patients with a less favorable course appear to either clear the virus poorly or to have a greater level of virus production [24]. It was recently demonstrated that positivity for anti-HBc alone in absence of HBsAg and anti-HCV is not rare in Japanese patients with HCC, which may indicate that HBV virus might be involved in so-called non-B HCC [25].

The entire nucleotide sequences of HBV genomes have been classified into 8 genotypes (A-H), with predominance of genotypes A and D in Western countries, and B and C in Southeast Asia and the Far East [26-29]. Several studies from the Far East evaluated the association between distinct genotypes and severity of liver disease. Genotype C was shown to be associated with the development of liver cirrhosis and HCC in Taiwan [30], China [31] and Japan [32], whereas genotype B was shown rarely to be associated with the development of HCC in China and Japan. In contrast, in Taiwan genotype B is the predominant type in patients with HCC who are younger than 35 years [30]. Another study from Taiwan showed that patients with genotype C had a greater tumor recurrence rate after curative resection of HCC compared with those with genotype B [33]. It was also shown that the likelihood of presence of T1762/A1764 mutations in the basal core promoter parallels the progression of liver disease, and that this mutation is found more frequent in HBV genotype C than B patients [34]. PreS deletions were shown to be more frequent in patients with HBV genotype C, and associated with more advanced disease such as liver cirrhosis and hepatocellular carcinoma [35].

#### Hepatitis C

Hepatitis C is also a major public health problem. There are more than 170 million people infected worldwide [36]. Approximately 80% of HCV infected patients develop chronic hepatitis C. About 20% of these patients will develop severe chronic hepatitis C and cirrhosis, which becomes detectable in the second and third decade after infection. The natural history of chronic hepatitis C infection is characterized by a predominantly asymptomatic course and a variable clinical outcome. For these reasons it is difficult to define the rate of progression to cirrhosis and HCC. The risk of cirrhosis in chronic hepatitis C is less than 10% in women infected at a young age and >30% in men infected after the age of 40 over a 20 year period [37,38]. Five prospective studies from Europe and the US have shown that during the first 10–15 years after initial infection, liver cancer is a rare occurrence [39-43]. In patients with hepatitis C, there is an increased risk of HCC coinciding with the establishment of cirrhosis with yearly incidence between 3–8% [6,7,44-47]. In Japan, the mean interval between infection and development of HCC is 30 years [48]. A study from the US shows a long time lag (mean 28 years, range 8–42) between transfusion-associated hepatitis and development of HCC [49].

There is conflicting information on the relationship between HCV genotype and progression to HCC in longitudinal studies. It is suggested by some authors that genotype 1b (most prevalent in Europe and Japan) is associated with a higher incidence of HCC than infection with other genotypes [50,51]. In other studies, however, this was not observed [52,53].

#### Coinfection of HBV and HCV

Both HBV and HCV are transmitted parenterally and coinfection is not uncommon in intravenous drug users and in countries with a high prevalence of HBV [54]. Coinfection of HBV and HCV seems to result in more severe liver disease than either infection alone [55]. The risk of developing HCC in subjects with both infections has been investigated in a meta-analysis of 32 epidemiological studies between 1993 and 1997 [56]. The odds ratio for development of HCC in HBsAg positive, anti-HCV/HCV RNA negative subjects was 20.4; in HBsAg negative, anti-HCV/HCV RNA positive subjects 23.6; and subjects positive for both markers 135. These data suggest a more than additive but less than multiplicative effect of HBV and HCV coinfection on the relative risk for HCC. The viruses may act through common as well as different pathways in the carcinogenic process.

It has been reported that HBV DNA is still present after seroconversion of HBsAg in patients with hepatitis B. Several reports indicate that prior HBV infection, characterized by presence of anti-HBc, affects the development of HCC in patients infected with HCV [57-59]. Given these data, in patients with chronic HCV infection, serologic markers of past HBV infection should be checked, not just HBsAg. Other authors, however, were not able to document any adverse event of occult HBV infection on the clinicopathologic course of chronic HCV infection [60].

In case of coinfection with HBV (whether active or past), a more aggressive surveillance to detect early HCC could be suggested [61]. However, to date screening and surveillance programs have not demonstrated a significant survival benefit.

In view of the role of HBV as cofactor in the development of HCV related cirrhosis and HCC, vaccination of patients with chronic hepatitis C against HBV has been advocated with the presumption of avoiding additional liver injury [62,63].

#### Coinfection of HBV and HDV

Verme *et al *[11] suggested that HBsAg positive patients with HDV superinfection develop cirrhosis and HCC at an earlier stage (mean age 48 year) than HBsAg carriers without HDV infection (mean age 62 years).

#### Coinfection of HBV and HCV with HIV

Coinfection of HBV and HCV with HIV is common because these diseases share the same routes of transmission. Recently a series of HCC in HIV-HCV coinfected patients was published, indicating an unusually rapid development of HCC in these patients [64]. This is not surprising, as chronic hepatitis C is more aggressive in HIV positive subjects, leading to cirrhosis and end-stage liver disease in a shorter period of time [65].

#### Coinfection of HCV and *S. mansoni*

An Egyptian study showed that Schistosoma infection increased the risk of HCC, only in the presence of HCV, whereas isolated *S. mansoni *infection does not [66].

#### Role of alcohol consumption in HBV or HCV infection

Reports suggest that HBV and ethanol act synergistically to promote HCC [67,68]. Habitual heavy drinking was reported to be a significant risk factor for HCC in patients with HCV-related liver cirrhosis by multiple logistic regression analysis [57]. A recent study showed synergism between alcohol drinking and HBV or HCV infection, with approximately a twofold increase in the odds ratio for each hepatitis virus infection for drinkers' > 60 g/d, with a more than additive but less than multiplicative risk [69]. Although two case-control studies did not show a relationship of alcohol consumption with the occurrence of HCC [70,71], another case-control study found a positive interaction between HBsAg positivity and HCV RNA positivity and heavy alcohol intake in the development of HCC [72]. Furthermore, Hassan et al. [73] showed synergistic interaction (more than additive) between heavy alcohol consumption ≥ 80 ml/d and chronic HBV or HCV infection (odds ratio 53.9) and insulin or non-insulin dependent diabetes mellitus (odds ratio 9.9).

### Incidence of HBV- and HCV-related HCC worldwide

Chronic hepatitis B and C infection are responsible for the great majority of cases of HCC worldwide [9]. They also account for the peculiar geographical distribution of the tumor. The relative frequencies of HBV and/or HCV related HCC in the world is illustrated in Table 1[17,72,74-93]. The worldwide incidence of HCC varies and is predominantly related to the regional prevalence of chronic viral hepatitis and its associated chronic liver disease and cirrhosis. Aflatoxin intake has a role in the genesis of HCC only in patients who have pre-existing chronic hepatitis B [84].

**Table 1:** Relative frequencies of HBV and HCV related HCC in the world

**Author [reference]**	**Country**	**Era**	**Sample size**	**HBsAg + (%)**	**Anti-HCV + (%)**	**HBsAg/anti HCV + (%)**	**Other (%)**
Chen, 1990 [74]	Taiwan	NR	66	**35 (53.0)**	15 (22.7)	7 (10.6)	9 (13.6)
Chuang, 1991 [75]	Taiwan	NR	128	**87 (68.0)**	13 (10.1)	12 (9.4)	16 (12.5)
Lee, 1992 [76]	Taiwan	NR	326	**233 (71.5)**	31 (9.5)	10 (3.1)	52 (15.9)
Jeng, 1991 [77]	Taiwan	NR	129	**62 (48.1)**	29 (22.5)	19 (14.7)	19 (14.7)
Leung, 1992 [78]	Hong Kong	1986–90	424	**341 (80.3)**	16 (3.8)	15 (4.0)	52 (12.3)
Nishioka, 1990 [79]	Japan	NR	180	64 (35.6)	**80 (44.4)**	11 (6.1)	25 (13.9)
Saito, 1990 [80]	Japan	NR	253	49 (19.4)	**136 (53.8)**	2 (0.8)	66 (26.1)
Kiyosawa, 1990 [17]	Japan	1958–89	83	19 (22.9)	**51 (61.4)**	10 (12.0)	3 (3.6)
Hassan, 2001 [81]	Egypt	1995–96	33	5 (15.2)	**25 (75.8)**	NR	NR
Kew, 1990 [82]	South Africa	NR	380	137 (36.1)	63 (16.6)	47 (12.4)	127 (33.4)
Yu, 1990 [83]	USA	1984–89	58	22 (37.9)	**36 (62.1)**	NR	NR
Di Bisceglie, 1991 [84]	USA	1987–88	99	7 (7)	12 (12)	1 (1)	**79 (79)**
Hadziyannis, 1995 [85]	Greece	1991–92	65	**33 (50.8)**	5 (7.6)	3 (4.5)	23 (38.3)
Colombo, 1989 [86]	Italy	1975–88	132	19 (14.4)	**64 (48.5)**	22 (16.7)	27 (20.5)
Levrero, 1991 [87]	Italy	1980–88	167	38 (22.8)	**82 (49.1)**	15 (9.0)	32 (19.2)
Simonetti, 1992 [88]	Italy	1982–88	212	15 (7.1)	**133 (62.7)**	18 (8.5)	46 (21.7)
Donato, 1997 [72]	Italy	1995–96	172	37 (21.5)	65 (37.8)	4 (2.3)	66 (38.4)
Stroffolini, 1998 [89]	Italy	1996–97	1083	125 (11.5)	**771 (71.2)**	55 (5.1)	132 (12.2)
Bruix, 1989 [90]	Spain	NR	96	4 (4.2)	**67 (69.8)**	5 (5.2)	20 (20.8)
Nalpas, 1991 [91]	France	1982–89	55	3 (5.5)	**28 (50.9)**	9 (16.3)	15 (27.3)
Van Roey, 2000 [92]	Belgium	90s	154	37 (24.0)	**62 (40.0)**	NR	55 (36.0)
Haydon, 1997 [93]	UK	1985–94	80	13 (16.3)	22 (27.5)	2 (2.5)	**43 (53.8)**

NR: not reported; Bold: predominant cause

In the Far East and sub-Saharan Africa, where HBV is highly endemic, HBV is the main cause of HCC.

In areas with an intermediate rate of liver tumors such as Southern Europe, Egypt and Japan, HCV is the predominant cause of HCC. Here HCC is mostly discovered at an older age in patients with longstanding cirrhosis due to HCV.

In regions with a low incidence of HCC such as Northern Europe and the United States, HCC related to HCV or HBV infection are found in a minority of cases and the tumor is often related to other factors such as alcoholic liver disease. In these low endemic areas, HCC is usually discovered at an older age in patients with longstanding cirrhosis due to alcohol abuse [72]. In France, ethanol is still the leading cause of cirrhosis and was responsible for 60% of all HCC causes during the last decade [8].

### Time trends in the incidence of HCC

An important epidemiological fact is the rising incidence of HCC in developed countries during the last two decades [79,89,95,99](Table 2).

**Table 2:** Time trends on the incidence of HCC in the world

**Author [reference]**	**Country**	**Number/100,000 era 1**	**Number/100,000 era 2**
El Serag, 1999 [95]	USA	1976–80: 1.4	1991–95: 2.4
El Serag, 2000 [96]	USA	1993–95: 2.3	1996–98: 7.0
Benhamiche, 1998 [97] (men)	France	1976–79: 7.5	1992–95: 10.2
Stroffolini, 1998 [89]	Italy	1969: 4.8	1994: 10.9
Law, 2000 [98] (men)	Australia	1983–85: 2.1	1995–96: 4.0
Nishioka, 1991 [79]	Japan	1968–77: 9.5	1984–85: 16.0
Yoshizawa, 2002 [99]	Japan	1980: ca 10	2000: ca 40

In Japan, the HCC-related mortality rate has sharply increased since 1975 from 10/100,000 to almost 40/100,000 in 2000 [99]. An analysis of the Shinshu University Hospital (Japan) showed a change in etiology of the HCC [100]. Whereas in the 1971–1980 decade, hepatitis B was the predominant cause of HCC, in the 1991–1995 period hepatitis C was largely predominant (Table 3). However, the total numbers of yearly deaths because of HCC in HBsAg carriers' stays constant, approximately 10% in the survey conducted in 1995. The rapid increase of mortality due to HCC in Japan is mainly attributable (ca 80%) to persistent infection with HCV [99]. The hepatitis C epidemic in Japan originated due to intravenous drug use by the young generation after World War II during the late 40s and early 50s. It spread in the general population due to remunerated blood donors. Abrogation of paid blood donation in 1968, exclusion of blood units contaminated with HBV in 1973 and HCV in 1989 decreased the risk of posttransfusion hepatitis from > 50% in the 60s to almost zero at present. The incidence of HCV in Japan is decreasing. As the interval between the time of the initial infection with the hepatitis C virus and the development of HCC is 30 years [79], the growing incidence of HCC in Japan is expected to reach a plateau around the year 2015, and then to decrease [99].

**Table 3:** Changing causes of HCC in Japan, 1971–95

**Author [reference]**	**Era**	**Sample size**	**HBsAg + (%)**	**Anti-HCV + (%)**	**HBsAg/anti HCV + (%)**	**Other (%)**
Kiyosawa, 1992 [100]	1971–80	112	**60 (54%)**	38 (34%)	5 (4%)	9 (8%)
	1981–90	267	82 (31%)	**159 (59%)**	4 (2%)	22 (8%)
	1991–95	162	21 (13%)	**126 (78%)**	5 (3%)	10 (6%)

Bold: predominant cause

Also in Italy the mortality rate of HCC is rising [89] from 4.8/100,000 in 1969 to 10.9/100,000 in 1994, reflecting the large cohort of subjects infected with HCV through iatrogenic route during the 50s and 60s when glass syringes were commonly used for medical treatment. Likewise in Australia, France and the United States of America (US) the HCC mortality is increasing, most probably because people infected with HCV have grown old and reach the cancer-bearing age [95-98]. In the US, an increase of about 80% in the incidence of HCC over the past 20–30 years is described, it is estimated that approximately 15,000 new cases occur each year. Also in France the incidence of HCC is steadily and markedly increased, the estimated number being about 4,000 per year [101].

Although the prevalence of HCV is declining in developed countries because of the decline in incidence in the 90s, the number of persons infected for ≥ 20 years is expected to increase substantially before peaking in 2015 [102].

Analysis of long-term serial HCV samples from the US and Japan suggest that HCV was introduced into the US population around 100 years ago and widely disseminated in the 1960s. In contrast, HCV was introduced in Japan > 100 years ago and widely disseminated in the 1930s and 40s. The HCV genotype 1b population in Japan started to decrease around 1995 whereas HCV genotype 1a in the US is still growing exponentially. It is predicted that an increased HCC prevalence will occur in the US over the next two to three decades [103].

The reasons advocated for explaining the increased incidence of HCC are the increased rate of HCV infection and an improvement of the clinical management of cirrhotic patients. Enhancing the survival of patients with advanced cirrhosis leads to an increased incidence of HCC. In fact, a decade ago, most of the deaths in cirrhotic patients were due to digestive hemorrhage or bacterial infections, two conditions that are now efficiently prevented and cured [104]. Therefore, HCC has become the leading cause of death in patients with cirrhosis.

### Screening tests for HCC in patients with chronic viral hepatitis

Despite knowledge of the risk factors for HCC, screening of HCC is controversial, as there have been no randomized controlled studies demonstrating the efficacy of screening for HCC. As HCC mostly occurs in patients with cirrhosis, or at least advanced fibrosis, most studies have been performed in these patients at risk. The most frequently used tests have been serum alfa-fetoprotein (AFP) and hepatic ultrasound (US).

There is one non randomized prospective cohort study suggesting that HCC was detected earlier and was more often resectable in patients who had twice yearly screening with serum AFP and hepatic US than in patients who had usual care [105].

Twenty-four studies, which included patients with chronic hepatitis B or C or both, addressed the sensitivities and specificities of screening tests [106].

Serum AFP for detection of HCC was evaluated in 19 studies. They were relatively consistent in showing that the sensitivity of serum AFP for detecting HCC increases from very low levels to moderately high levels of 60 to 80% as the threshold value decreased from 400 to 10 ng/mL, with corresponding specificity decreasing from 100 to 70–90%. A threshold between 10 and 19 ng/mL seems most appropriate as sensitivity usually is moderately high (45 to 100%), with a specificity of 70 to 90%. It has been shown that AFP is not always specific for HCC and titers can increase with flares of active hepatitis [107].

Seven studies evaluated screening with US, reporting high specificity of 95–100%, but variable sensitivity, varying from 11–99% [94].

A surveillance study combining US and AFP in 1,125 patients with HCV, HBV or both, reported a sensitivity of 100% when using a serum AFP > 10 ng/mL together with US, compared with a sensitivity of 75% using only AFP > 10 ng/mL and a sensitivity of 87% when using US alone [108].

Computed tomography and magnetic resonance imaging have a high sensitivity and specificity in detecting HCC, but are too expensive to be used in surveillance [1].

The surveillance intervals studied varied from 3 to 12 months. In a study of patients with hepatitis B, the most rapidly growing tumor increased from 1 to 3 cm in 5 months [109]. The ideal time for re-screening has not been identified. Some investigators suggest a 4–5 month interval, others have suggested that a 6-month interval may be most appropriate [109,110]. It is suggested that in case of concomitant HBV and HCV infection serum AFP levels should be obtained every 3 months, and that persistent AFP levels should prompt an aggressive imaging search for HCC [61].

It can be concluded that screening patients with advanced chronic hepatitis B or C with AFP and US may improve detection of HCC, but further studies are needed whether screening improves clinical outcomes.

## Pathogenesis of hepatitis B and C-induced hepatocellular carcinoma

### Introduction

Epidemiologic data indicate that chronic hepatitis B and C are independent risk factors for development of HCC [7,16]. Furthermore, animal models confirm the oncogenic potential of HBV and HCV in the liver: transgenic mice for hepatitis B and C [110,111], and natural models such as the woodchuck infected with the woodchuck hepatitis virus, a hepadnavirus closely related to the HBV [112].

Carcinogenesis is believed to be a multistage process, occurring through a sequence of steps termed *initiation*, *promotion *and *progression*. This process evolves over several or many years. Tumor *initiation *begins in cells through mutations induced by exposure to carcinogens. DNA changes, maintained during successive cell divisions, activation of oncogenes and inactivation of suppressor genes lead to dysregulation of the cell division and to immortalization [113]. Tumor-initiated cells have a decreased responsiveness to both intercellular and intracellular signals that maintain normal cellular architecture and regulate homeostatic growth. Tumor *promotion *results in a further selective clonal expansion of initiated cells. During tumor *progression*, pre-malignant cells continue to develop progressive phenotypic changes and genomic instability (dysplasia), culminating as overt carcinoma [115].

More than 80% of HCC originate in cirrhotic livers. Macronodules (macroregenerative nodules and adenomatous hyperplasia), irregular hepatocyte regeneration, and some hyperplastic foci are considered as precancerous [116-119]. Large cell dysplasia and small cell dysplasia are considered to be risk factors for development of HCC [120-122].

HBV and HCV can be implicated in the development of HCC in an indirect way, through induction of inflammation, necrosis and chronic hepatocellular regeneration, or directly by means of viral proteins or, in the case of HBV, by creating insertional mutations by integration in the genome of the hepatocyte.

### Indirect carcinogenicity of HBV and HCV

In most patients with chronic hepatitis B and/or C the occurrence of HCC is preceded by a process of longstanding inflammation. It is probable that malignant transformation is related to continuous or recurring cycles of hepatocyte necrosis and regeneration [123]. The resulting accelerated cell turnover rate may act as a tumor promotor by increasing the probability of spontaneous mutations or damage to DNA by exogenous factors. The accelerated rate of cell division leaves less time for altered DNA to be repaired before the cell divides again, resulting in transmission of altered DNA to the daughter cells. In this way a series of mutations may accumulate in individual cells over time. This process can lead to focal uncontrolled liver cell growth and eventual malignant cell transformation [115,124]. Another mechanism of induction of malignant transformation is the generation of mutagenic reactive oxygen species as a result of the inflammatory process, such as nitric oxide (NO), superoxide anion (O_2_^-^), hydroxyl radical (OH•) and hydrogen peroxide (H_2_O_2_) [124].

Evidence for a causal role for chronic necro-inflammation is provided by transgenic mice into which HBV preS/S genes have been introduced. These mice overproduce pre S1 protein that accumulates in the endoplasmatic reticulum of hepatocytes, producing severe and prolonged injury to these cells, initiating a response characterized by inflammation, regenerative hyperplasia and transcriptional deregulation that progresses ultimately to neoplasia [125].

Patterns of gene expression in cirrhosis and hepatocellular carcinoma have recently been shown to be of value in predicting prognosis. Kim *et al *could identify, using the complementary DNA microarray, a 273-gene signature that distinguished high risk types of cirrhosis (hepatitis B, hepatitis C, hereditary hemochromatosis) from low risk types (autoimmune hepatitis, PBC, alcoholic liver diseases) [126]. The same 273-gene signature was present in samples from patients with proven HCC. A subset of 30 genes was most significantly altered in both the high risk types of cirrhosis and the HCC patients. The TACSTD1, a gene associated with HCC development in other studies, is a lead gene in this gene signature. Lee *et al *could identify a limited number of genes that accurately predicted survival in a series of 91 HCC patients [127]. The genes involved are implicated in cell proliferation and apoptosis, but also in ubiquitination and histone modification. Delpuech *et al *identified distinct patterns of gene expression according to the viral aetiology [128]. Finally, Hann *et al *could demonstrate the presence of antibodies to differentially expressed genes in hepatitis B and C, and this appeared to be linked with decreased survival [129]. These discoveries not only increase our insight in hepatocarcinogenesis, but may ultimately lead to the development of clinically valuable preneoplastic and prognostic blood markers.

### Direct carcinogenicity of HBV and HCV

#### Hepatitis B

A significant proportion of HBV-related HCCs arise in an otherwise normal liver, implicating that the virus can also be directly oncogenic [124].

It has been demonstrated that HBV integrates into the DNA of the host cells. This integration may dysregulate the control mechanisms on the cell cycle by chromosomal abnormalities, production of viral proteins or alteration of human genes and proto-oncogenes. It is, however, controversial whether viral integration plays an important role in the process leading to development of HCC. The hepadnaviral integration process appears to involve recombination mechanisms that do not preserve the viral genome sequence. Thus it is impossible for the viral integrant to function as a template for subsequent virus replication. Several studies suggest that DNA integration sites are at random and that integration occurs at random times during the course of a chronic viral infection [130,131]. HBV integration can be present in chronically infected liver tissue without evidence of HCC [132]. Non-neoplastic hepatocytes may have a similar pattern of rearrangement of viral sequences following integration into human DNA.

### Chromosomal DNA instability

Several studies have shown that HBV DNA integration enhances chromosomal instability. In many hepatic tumors large inverted duplication insertions, translocations and micro- and macrochromosomal deletions have been associated with HBV insertion [133-136]. These changes can result in loss of important cellular genes, sometimes involving tumor-suppressor genes and other genes involved in the regulation of regeneration and growth processes.

#### Trans-activation of cellular genes

HBV DNA may induce malignant transformation in another way.

Mammalian hepadnaviruses contain a gene (the HBX gene), of which the protein (HBX protein) can *trans*-activate several cellular promotors and upregulate their expression of different cellular and viral genes [137,138]. Integrated HBX, even when truncated, frequently encodes functionally active *trans*-activator proteins [139]. This protein has been shown to transform mouse fetal hepatocytes into a full malignant phenotype [140]. There are studies in transgenic mice with the HBX gene that developed multifocal areas of altered hepatocytes, adenomas and HCCs [110].

In contrast to mammalian hepadnaviruses associated with HCC, avian hepadnaviruses such as the duck hepatitis virus or heron hepatitis virus, lack the HBX gene and are not associated with HCC [123].

A gene that may be affected by the HBX gene is the p53 tumor suppression gene. This gene has been shown to play an important role in hepatocarcinogenesis. It is considered to negatively regulate the cell cycle. The HBX protein has been shown to complex p53 protein and to inhibit its function [141,142]. In a transgenic mouse model it was shown that HCC development correlates with p53 binding to HBX [143].

#### Oncogenes

It has been proposed that HBV acts as an insertion mutagen by integrating into the host genome and activating the cellular proto-oncogenes *c-myc*, *ras *and *c-fos *[144].

The preS2/S gene is integrated in most HCCs associated with HBV. When 3'-truncated it generates a truncated protein that is oncogenic by *trans*-activating proto-oncogenes *c-myc *and *c-fos *[145].

#### Growth factors

Growth factors and their receptors function as positive or negative modulators of cell proliferation and differentiation. Insulin-like growth factor-II and transforming growth factor-β expression correlate with HBX protein expression in animal models [146,147], suggesting *trans*-activation of these growth factors facilitating tumor formation.

#### Role of PreS mutations

PreS deletion mutants accelerate the storage of large envelope proteins in hepatocyte cytoplasm, which could induce cytotoxic effects toward the development of end-stage liver disease [148]. The accumulation of large envelope protein can activate cellular promoters by inducing endoplasmic reticulum stress [149]. Furthermore, pre-S1 sequences can stimulate the transcription of transforming growth factor α (TGFα). Coexpression of TGFα and HBsAg could accelerate hepatocellular carcinogenesis by stimulation of hepatocyte proliferation [150].

#### Allelic loss of chromosome 4q

Allelic loss of chromosome 4q is one of the most frequent genetic aberrations found in HCC. It was found to be associated with HBV-related hepatocarcinogenesis, probably by inactivation of a putative tumor suppressor gene included in it [151].

### Hepatitis C

In contrast to HBV, HCV is an RNA virus that lacks a reverse-transcriptase enzyme and cannot integrate into the host genome. Thus, insertional mutagenesis can be excluded as a pathogenic mechanism for the development of HCC associated with chronic HCV infection. The molecular pathogenetic mechanisms by which HCV contributes to cell transformation remain unclear.

One possibility is that the development of HCC is simply related to chronic necro-inflammatory liver disease. Overall, 97% of patients with HCV markers and HCC have cirrhosis [152,153], and most of the remainder develop HCC in the presence of chronic hepatitis.

An alternative mechanism of HCV-induced hepatocarcinogenesis may be that HCV has a direct oncogenic action. Viral replication might cause inappropriate expression of two growth factors that may be implicated in hepatic carcinogenesis: transforming growth factor-α and insulin-like growth factor II [154,155].

The non-structural HCV protein *NS3 *has both protease and helicase activity. HCV may therefore induce genomic instability and favor mutations through its helicase activity [156]. The protein also has an activity similar to protein kinase A, and could disturb cellular homeostasis [157].

The *HCV envelope protein E2 *and the non-structural protein *NS5A *inhibit RNA-dependent protein kinase, key mediator of the antiviral, antiproliferative and anti-oncogenic effect of interferon [158-160].

The *HCV core protein *has characteristics that imply that this protein could function as a gene-regulator [161,162]. The presence of the protein in transgenic mice can induce HCC [111]. After mutation, the HCV core protein can also inhibit tumor suppressor genes such as p53, as has been demonstrated in hepatic oncogenesis [163-165]. It has recently been shown that the HCV core protein induces nuclear factor κB (NF-κB), thereby suppressing TNF-α-induced apoptosis [166]. This anti-apoptosis may be a mechanism by which HCV leads to viral persistence and possibly to hepatocarcinogenesis.

### Prevention of hepatocellular carcinoma caused by viral hepatitis

#### Primary prevention

The most effective tool to prevent HCC is avoidance of the risk factors such as viral infection by HBV or HCV. Any action diminishing the potential transmission of contaminated blood products (uncontrolled blood transfusion, needle sharing, invasive procedures without proper health standards) will decrease the likelihood of viral spread.

The major advance has come from the availability of an effective vaccine that protects against HBV.

In 1969, Taiwan was an hyperendemic area of HBV infection with a high rate of HBsAg positivity, 19% of the population being infected before the fourth decade of life. In 1976, HBsAg prevalence was > 80% in HCC in Taiwan [167]. In 1984 a program to control cirrhosis and HCC began. All neonates born to HBsAg positive mothers were given hepatitis B vaccine in order to counter perinatal infection. In 1986 all neonates were included in the program. As a consequence, there was a decrease in HBsAg positivity in six-year-olds from 10.6% in 1983–1984 to 0.8% in 1993–1994. There was a parallel decline in incidence of childhood HCC (6–14 years old), in the cohort born between 1980 and 1984. The incidence of liver cancer in children between 6 and 14 years old decreased to zero for children born in 1986 and 1987 [168]. The decline of HCC in children after universal vaccination can be considered as an early indicator of the effectiveness of vaccination in reducing the rate of HCC. Since the incidence of HCC in Taiwan peaks in the sixth decade of life, it may take 40 years or longer to see an overall decrease in the rate of HCC as a result of the vaccination program. Vaccination against HBV should become a health priority together with the promotion of adequate health standards.

Unfortunately, there is no vaccine against HCV. Up to now, the only effective method to prevent its transmission is the avoidance of contamination with infective blood products.

## Prevention of HCC in patients with previously acquired risk

### Introduction

Chronic viral carriage is one of the main risk factors for the development of HCC. Effective antiviral treatments have been developed in recent years and this has changed the management of viral infection.

Interferon-alfa is still considered the reference therapy for HBeAg positive chronic hepatitis B. However, its efficacy is limited, with seroconversion from anti-HBe negative to anti-HBe positive in up to 40%. Only <10% of patients become HBsAg negative [169]. Other possible treatments are antiviral drugs such as lamivudine and adefovir dipivoxil [12].

For the treatment of chronic hepatitis C, interferon-alfa monotherapy yielded only limited response. Combination with ribavirin led to a significant increase in sustained viral response to about 40% in treatment-naïve patients [170,171]. Recently, the combination of peginterferon-alfa and ribavirin improved the sustained viral response rate to nearly 60% in treatment-naïve patients [172,173], and is now considered the reference treatment.

It is under debate whether interferon-alfa-based treatments are effective in declining the incidence of HCC in chronic hepatitis B and C.

### Anti-oncogenic effects of interferon-alfa

HCC prevention by interferon-alfa might be the result of several direct or indirect mechanisms. Interferon has an antiproliferative and pro-apoptotic effect [174]. Interferon inhibits the expression of the *c-myc *oncogene and induces the expression of anti-proliferative factors and tumor suppressor genes [175-177]. In experimental animal models, the anti-neoplastic potential of interferon was demonstrated in already established tumors. In a transgenic mouse model it was demonstrated that early and prolonged administration of interferon diminished the severity of preneoplastic lesions and slowed down the development of HCC [178]. Interferon-alfa also could indirectly reduce the oncogenic risk by inhibition of synthesis of viral proteins which potentially dysregulate the cell cycle, and by enhancing the immune system eliminating not only infected hepatocytes but also initiated or fully malignant cells. Furthermore, interferon-alfa has an antifibrotic and anti-angiogenetic effect, which could also have an influence on tumor development [179].

### Interferon and antiviral treatment

#### Noncirrhotics

In patients with chronic hepatitis B, clearance of the HBeAg after treatment with interferon-alfa is associated with improved clinical outcome in terms of survival and development of complications of cirrhosis [180]. Another study confirmed these results and showed a reduction of incidence of HCC in the responders [181]. As most of these patients were non-cirrhotics at entry of the study, the prophylactic effect of interferon on development of HCC can be explained by prevention of cirrhosis development. In Chinese patients with chronic hepatitis B infection, however, interferon-alfa was of no long-term benefit in inducing HBeAg conversion, or in the prevention of HCC and other cirrhosis-related complications [182].

#### Cirrhotics

Seven studies investigated the possible effect of interferon treatment on development of HCC in patients with already established cirrhosis [183-189] (Table 4). A meta-analysis was performed on these studies [190]. Interferon seemingly decreased the rate of HCC in all trials, while a significant difference was observed in 2 studies [183,186]. Virologic response was strongly associated with reduced risk for HCC in the studies of Oon [183] and Mazzella [184], suggesting that arrest of viral replication is a critical factor. Subgroup analysis in relation to ethnic origin of patients (European, Oriental) showed no preventive effect of interferon on the development of HCC in the European patients [190].

**Table 4:** Studies of treatment with interferon-α for prevention of HCC in patients with hepatitis B-related cirrhosis

**Author [reference]**	**Country**	**Type of study**	**Interferon regimen (duration in weeks)**	**Follow-up (range) in months**	**Sample size**	**Rate of HCC (n/n)**	**Significance**
Oon, 1992 [183]	Singapore	NRCT, P	10 MU daily, 10 days/month (12)	12 (12–60)	T 600 C 180	T: 0/600 (0%) C: 10/180 (5.6%)	Significant
Mazzella, 1996 [184]	Italy	NRCT, P	10 MU tiw (26)	49 (12–119)	T 34 C 28	T: 2/34 (5.9%) C: 4/28 (14.3%)	Not significant
Fattovich, 1997 [185]	Europe	NRCT, P	≥ 300 MU (12–52)	84 (80–92)	T 40 C 50	T: 3/40 (7.5%) C: 4/50 (8.0%)	Not significant
Ikeda, 1998 [186]	Japan	NRCT, P	12 MU/wk (26)	84 (6–168)	T 94 C 219	T: 10/94 (10.6%) C: 51/219 (23.3%)	Significant
IHCSG, 1998 [187]	Argentina, Germany, Italy, Saudi Arabia	NRCT, P	9–30 MU/wk for 3–30 months	(36–250)	T 49 C 97	T: 8/49 (16.3%) C: 18/97 (18.6%)	Not significant
Benvegnù, 1998 [188]	Italy	NRCT, P	6–10 MU (20–26)	72	T 10 C 18	T: 0/10 (0%) C: 4/18 (22.2%)	Not significant
Di Marco, 1999 [189]	Italy	NRCT, P	655 MU	93 (6–180)	T 26 C 60	T: 2/26 (7.7%) C: 6/60 (10%)	NR

NRCT: non-randomized controlled trial

P: prospective

T: treated

C: controls

MU: million units

NR: not reported

It should be noted that the studies are very heterogeneous and that none of them were randomized controlled trials, so that the results should be interpreted with caution.

A recent study showed a significant reduction of the risk of HCC in patients with chronic hepatitis B and advanced fibrosis or cirrhosis, treated with lamivudine for a maximum of five years, compared to placebo [191].

### Interferon treatment in HCV patients and HCC prevention

#### Noncirrhotics

Three studies assessed whether interferon treatment prevents the development of HCC in noncirrhotic patients with chronic hepatitis C [192-194] comprising 3,798 noncirrhotic patients treated with interferon-alfa monotherapy. Pooled together, the incidence of HCC was 60/2,532 (2.37%) in sustained virological responders and 76/1,266 (5.29%) in nonresponders. In a study of 291 noncirrhotic patients with chronic hepatitis C who were nonresponders to interferon therapy and followed for 6–117 months after therapy, the incidence of HCC was significantly lower in patients who received > 500 MU of interferon. Patients with a transient response (i.e. relapse after end of treatment) had a significant lower rate of HCC development (4/166 = 2.4%) than nonresponders (12/125 = 9.6%) [195].

This anti-oncogenic benefit can presumably be explained by an arrest or slowing down of the cirrhogenic process.

#### Cirrhotics

The findings of 13 studies of interferon treatment and development of HCC in HCV-infected patients with compensated cirrhosis are summarized in Table 5[45,184-186,194,196-204]. Only 3 studies were randomized [199,201,202,204], the remainders were observational cohort studies. Statistical combination of data is not possible because of different definitions of response (biochemical, virological), different dose schedules for interferon and different duration of follow-up. All studies showed a lower risk for development of HCC in the interferon-treated patients, suggesting that interferon may prevent HCC in compensated cirrhosis caused by hepatitis C. The overall result was largely influenced by three Japanese studies [194,198,201,202], which had the highest incidence of HCC in untreated patients (5–6% per year). This may be explained by intensiveness of the screening programs, but also by genetic, environmental or viral factors. Four European studies failed to document a significant reduction in risk of developing HCC [45,196,199]. In the studies of Fattovich *et al *[196] and Bruno *et al *[45], interferon-alfa treatment showed a decrease in incidence of HCC in univariate analysis. However, this was not present in multivariate analysis. In the study of Fattovich [196], a very low natural incidence of HCC was observed, rendering difficult to show a significant decrease. The prospective randomized controlled trial of Valla *et al *[199] also failed to show a significant effect of interferon treatment on the development of HCC. However, the number of patients in this study was limited and the follow-up relatively short. Also a recently published randomized controlled study from Italy comprising 51 interferon-treated and 71 untreated patients with compensated hepatitis C-cirrhosis, failed to demonstrate any reduced risk in development of HCC after a mean follow-up of 96.5 months [204].

**Table 5:** Studies on treatment with interferon-α for prevention of HCC in patients with HCV-related cirrhosis

**Author [reference]**	**Country**	**Type of study**	**Interferon regimen (duration in weeks)**	**Follow-up (range) in months**	**Sample size**	**Rate of HCC (n/n)**	**Significance**
Mazzella, 1996 [184]	Italy	NRCT, P	3 MU tiw (52)	32 (12–71)	T 193 C 91	T: 5/193 (2.6%) C: 9/91 (9.9%)	Significant
Fattovich, 1997 [196]	Europe	NRCT, P	≥ 200 MU	60 (1–153)	T 193 C 136	T: 7/193 (3.6%) C: 16/136 (11.8%)	Not significant
Bruno, 1997 [45]	Italy	NRCT, P	6 MU tiw (26)	68 (60–84)	T 82 C 81	T: 6/82 (7.3%) C: 14/81 (17.3%)	Not significant
Serfaty, 1998 [197]	France	NRCT, P	3 MU tiw (48)	40 (6–72)	T 59 C 44	T: 2/59 (3.4%) C: 9/44 (20.1%)	Significant
IHCSG, 1998 [187]	Argentina, Germany, Italy, Saudi Arabia	NRCT, R	9–30 MU/wk (3–30 months)	(36–250)	T 232 C 259	T: 2/232 (0.9%) C: 48/259 (18.5%)	Significant
Imai, 1998 [198]	Japan	NRCT, R	480 MU (26)	48 (3–65)	T 32 C 20	T: 8/32 (25%) C: 7/20 (35%)	Significant
Benvegnù, 1998 [188]	Italy	NRCT, P	3–6 MU tiw (26–52)	72	T 75 C 77	T: 4/75 (5.3%) C: 20/77 (26.0%)	Significant
Valla, 1999 [199]	France	RCT	3 MU TIW (48)	40 (37–53)	T 47 C 52	T: 5/47 (10.6%) C: 9/52 (17.3%)	Not significant
Yoshida, 1999 [194]	Japan	NRCT, R	480 MU (23)	52	T 230 C 107	T: 33/230 (14.3%) C: 29/107 (27.1%)	NR
Okanoue, 1999 [200]	Japan	NRCT, R	3–10 MU qd or tiw (16–24)	1–7 years	T 40 C 55	T: 7/40 (17.5%) C: 22/55 (40.0%)	NR
Nishiguchi, 1995/2001 [201,202]	Japan	RCT	6 MU tiw (12–24)	104 (31–110)	T 45 C 45	T: 12/45 (26.7%) C: 33/45 (73.3%)	Significant
Gramenzi, 2001 [203]	Italy	RCT, P	741 MU	72	T 72 C 72	T: 6/72 (8.3%) C: 19/72 (26.4%)	Significant
Testino, 2002 [204]	Italy	RCT	3 MU tiw (52)	96.5 ± 18	T 51 C 71	T: 15.51 (29.4%) C: 24/71 (33.8%)	Not significant

NRCT: non-randomized controlled trial

RCT: randomized controlled trial

P: prospective

R: retrospective

NR: not reported

T: treated

C: controls

MU: million units

In most studies, virological and/or biochemical response are associated with a lower risk of development of HCC, which is less clear in nonresponders. In the study of Imai et al. [198], patients with sustained biochemical response after interferon therapy were at low risk for development of HCC (risk ratio versus controls 0.06; 0.95 in nonresponders). Also in the study of Mazzella [184], a statistically significant effect of interferon treatment was demonstrated when biochemical responders were compared with controls but not when compared with nonresponders. In the study of Benvegnù *et al *[188], the beneficial effect of interferon treatment on development of HCC was independent of the type of response. In the study of Yoshida *et al *[194] the risk for HCC was reduced especially among patients with sustained virological but also merely biochemical response that tested positive for HCV RNA. Okanoue *et al *[200] studied 1,148 patients with chronic hepatitis C treated with interferon-alfa, 40 of them having cirrhosis (fibrosis stage F4). They were followed for 1–7 years after therapy. The cumulative incidence of HCC was significantly decreased in sustained biochemical responders, compared to nonresponders and transient responders, in patients with stage F2 fibrosis, but not in the more advanced stages F3 and F4. In the study of Testino *et al *[204] HCC did also develop in sustained biochemical responders. Tanaka *et al *[205], however, demonstrated in 55 patients with HCV-cirrhosis that long-term administration of interferon prevented HCC in those with biochemical and virological response, whereas HCC only appeared in nonresponders.

The mechanisms by which an interferon treatment might reduce the risk of HCC development in cirrhosis caused by HCV independent of virological response remains speculative. Maintenance of serum transaminases at low levels may protect against the development of HCC as hepatocyte necrosis, cell damage and increase in hepatocyte replication result in increased DNA damage, influencing hepatocarcinogenesis. Other possible mechanisms for prevention of HCC are the direct and indirect effects of interferon. It is, however, perplexing that only 6 or 12 months of therapy can produce this benefit without virological response. Because of potential biases in the published trials it is premature to advocate the use of interferon as established therapy in HCV infected patients with cirrhosis to prevent HCC. Prospective randomized controlled trials should reproduce the findings in large numbers of patients before a definitive conclusion on the long term effects of interferon in HCV cirrhosis can be established.

It must also be realized that a sustained virological response to interferon-alfa monotherapy can be obtained only in 0–8% of patients with cirrhosis [206-208]. New treatments are now available for chronic hepatitis C, which are more performant in difficultly to treat cases as patients with cirrhosis. The combination of interferon-alfa and ribavirin results in a sustained virologic response in up to 25% of cirrhotics due to hepatitis C [207]. A sustained virologic response of 32% was reported after peginterferon-alfa-2a monotherapy [207] and of 43% after combination of peginterferon-alfa2a and ribavirin [173]. It should be investigated in prospective trials, taking into account the sustained virological and biochemical responses if these more performant treatment regimens will also influence favorably the incidence of HCC, as no data on the long-term effects of these treatments are available up to now.

### Secondary prevention

A few sudies focus on the possible role of intereron in the secondary prevention of HCC recurrence in patients with chronic hepatitis B and C after curative resection or ablation.

Ikeda *et al *[209] showed that interferon prevented HCC recurrence after complete resection or ablation of the primary tumor depending on the clearance of HCV viremia. Kubo *et al *[210] reported a decreased recurrence after surgical resection independent of clearance of HCV or normalization of serum ALT. Another study demonstrated prevention of HCC recurrence after medical ablation therapy for primary tumors in hepatitis B but not in hepatitis C patients by the use of interferon-alfa [211].

## Conclusions

Chronic hepatitis B and C, mostly in the cirrhotic stage, are responsible for the majority of the hepatocellular carcinomas worldwide. The rising incidence in HCC in developed countries during the last two decades is due to the increasing rate of hepatitis C infection and improvement of the clinical management of cirrhosis. Vaccination against hepatitis B seems to protect against the development of HCC.

In patients with chronic hepatitis B or C, interferon alpha treatment in a noncirrhotic stage is protective for HCC development in responders, probably by prevention of cirrhosis development. When cirrhosis is already present, the protective affect is less clear. Further prospective long-term studies should be performed on the new treatments for chronic hepatitis B and C. Some studies also suggested a favourable effect of interferon alpha in the prevention of HCC recurrence in patients with chronic hepatitis B and C after curative resection or ablation.

## Competing interests

The author(s) declare that they have no competing interests.

## Authors' contributions

PPM participated in the literature search and was responsible for the redaction of the paper.

SMF participated in the redaction of the manuscript and critical review of the paper.

JLV participated in the literature search and finalizing of the lay-out of the paper.
